# Analysis of distillation characteristics via CFD (computational fluid dynamics) of Korean traditional ‘Sojutgori’ and study on structure for distillation efficiency enhancement

**DOI:** 10.1002/fsn3.3099

**Published:** 2022-10-09

**Authors:** Ung‐soo Kim, Jin‐ho Kim, Tae‐Wan Kim, Jung‐hoon Choi

**Affiliations:** ^1^ Korea Institute of Ceramic Engineering & Technology Icheon Korea; ^2^ Korea Food Research Institute Wanju Korea

**Keywords:** CFD, distillation, Onggi ceramic, sojutgori, South Korea's distiller

## Abstract

The design of the Korean traditional distiller ‘*sojutgori*’ was extracted as a digital sketch, and the internal fluid flow in the distillation process was tracked through computer simulation. Based on this, a new design was derived to improve distillation efficiency and its changes were researched. The ethanol particles vaporized inside the distiller were stagnated or their discharge was accelerated according to the magnitude and frequency of vortex. If the center is narrow and the fluid rotates, the vortex decreases or changes to a regular form. To effectively control the vortex, six simple models and two materialized models were designed and the optimal design was derived. When compared with the traditional distiller, the outlet fluid speed of the final design increased by 78% and the residence time dispersion of ethanol particles decreased by 39%. Furthermore, to suppress the temperature spread of fermented wash, a streamlined blade structure that can promote convection current was added. This structure had the effect of reducing the temperature spread of fermented wash by 57%. In addition, a reflux ring structure that can control the recondensed fermented wash caused by heat loss at the inner wall of the distiller was designed and applied. The reflux ring structure minimized the temperature change of the fermented wash and decreased temperature change by 23% compared to the condition without the reflux ring structure.

## Graphical abstract

This study suggests a new design for tracking and modernizing the flow of fluid that changes inside according to the shape and material of "Sojutgori", a traditional Korean distiller.

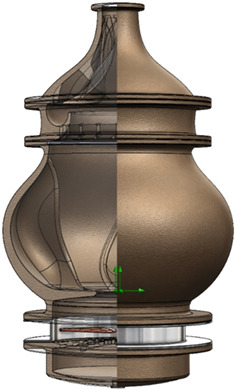

## 1 INTRODUCTION

The distillation technique developed by alchemists in the Middle East around the 10th century is known to have spread all across the world based on historical events such as the Crusades and the expansion of the Mongol Empire (Liebmann, 1956). The spread of distillation technique led to the development of distillers for the preparation of indigenous distilled liquors boasting unique tastes and flavors through innovations in the manufacturing technology and introduction of new techniques over time (Kockmann, 2014). The distillation technique also spread to Korea during the Mongol invasion in late Koryo dynasty (13th century) and resulted in the development of a Korean traditional distiller called *sojutgori*, which had been used actively for the production of Korean traditional distilled liquors until the first half of the 20th century (Kim, 2019). However, the consumption of diluted soju instead of distilled soju became mainstream through the Japanese occupation and the enactment of the Grain Management Act (1964) which restricted the use of grains in liquor manufacturing. Diluted soju refers to soju diluted to 15%–35% by adding water and additional flavoring additives after distilling high‐concentration alcohol with mass production in mind in the commercialized manufacturing process. Although the productivity of such diluted soju is increased, the inherent flavor is reduced during the dilution process. Due to such background of the times and industrialization, the technical development of, and interest in, the Korean traditional distiller *sojutgori* stagnated (Yum, 2011).

**FIGURE 1:**
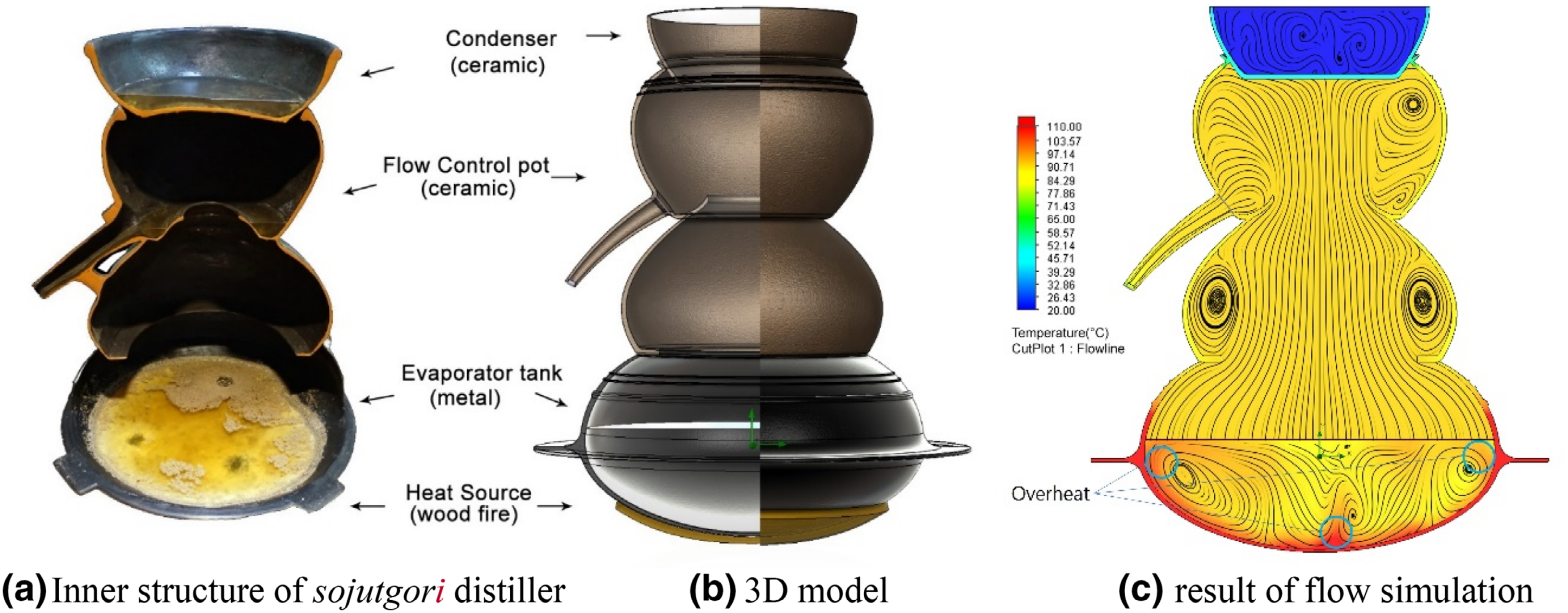
Analysis result and inner structure of the Korean traditional distiller *sojutgori*

*Sojutgori* has a structure that induces recondensation by heating fermented wash with direct fire using a container made of earthenware material and supplying cold water to the top of the container (Figure 1a). It is easy to use and maintain because the structure is simple and does not require complex additional devices for heating and cooling to induce phase change. The traditional distilled soju produced through distillation using such a *sojutgori* is known to have rich flavor and soft sensory quality. However, due to unstable temperature control and cooling structure, it is also known that factors that adversely affect taste such as raw material odor, wake odor, and burnt odor are strong (Kim, 2015). It has been reported that this is related to the temperature spread in the fermented wash due to the heating structure of the direct fire method and the component materials and shape of the distiller (Rankine, 1967). Studies are being conducted to examine the effects of the types of heat sources and the deformities and thermal properties of structures on the distillation efficiency. Many studies claimed that the computational fluid dynamics (CFD) method can be an effective tool (Khatir et al., 2012; Rousseau et al., 2006; Sharma et al., 2018; Xia & Sun, 2002). The CFD analysis is performed to convert Navier–Stokes equations, which are nonlinear partial differential equations describing fluid phenomena, to algebraic equations by discretization using the Finite Difference Method, Finite Element Method, Finite Volume Method, and so on, and then to solve and analyze fluid flow problems using the algorithm of numerical methods (Blocken et al., 2007; Griffiths & Boysan, 1996).

This study aims to analyze the correlation between the shape and material of the Korean traditional distiller *sojutgori*, which has not been modernized due to the background of the times and the change of market, and distillation efficiency through the CFD method, and to propose a new design that can improve distillation efficiency based on the analysis result. Specifically, a new design is derived through the fusion of modernized prior art and theory by tracking the fluid flow inside the traditional form of *sojutgori* and selecting elements governed by material and shape. This study is expected to suggest a direction for the modernization of the Korean traditional distiller *sojutgori*.

## 2 MATERIAL AND METHODS

To create a digital sketch of the *sojutgori* distiller, the regional *sojutgor* is stored in museums (Andong Soju Museum, National Folk Museum of Korea, and Andong Folk Museum) and information in various literature was referred to (Lee et al., 2017). After basic values were obtained through public information and optical measurements, they were computerized into three‐dimensional (3D) models using the Solidworks CAD software 2021 (Dassault Systems). Figure 1b shows the computerized 3D model of the *sojutgori*. Based on this 3D model, each fluid region was analyzed as a single phase separately for the fermented wash in liquid phase and ethanol vapor in gas phase. The boundary condition was set by the inlet mass flow rate based on the amount of extracted ethanol measured on the surface of fermented wash, and also by the outlet volume flow rate of ethanol vapor. For the liquid phase fermented wash flow, the temperature change and flow due to the heat source were tracked, and for the gas phase ethanol vapor, the turbulence, flow rate, and speed by the type of distiller were observed. The CFD analysis was conducted using the Solidworks Flow Simulation 2021 software (Dassault Systems). The material properties used for fluid analysis were set to ceramic (earthenware), metal (cast steel), ethanol vapor, water, and ethanol mass mixture.

The analysis of this study was conducted through three steps as follows.

In the first step, the temperature change caused by the energy that fermented wash received from the heat source and the flow and speed of fluid inside the liquid were tracked. The fermented wash area was set as one subfluid area, and 27‐point goals for tracking temperatures were set at the centers of three‐dimensionally evenly distributed grids. The number of goals that satisfy the condition of 78°C at which ethanol vaporizes was coupled with the inlet mass flow boundary condition of the fermented wash surface.

In the second step, the fluid flow of the ethanol vapor was observed based on the inlet mass flow in the fluid area of fermented wash. The gas area was also set as a subfluid area. To implement the recondensation effect of ethanol vapor due to heat loss at the outer boundary, 12 surface goals were set in the direction of height on the bulkhead comprising the gas area. Then the ethanol vapor loss was calculated by setting the outlet volume flow boundary condition in the area where the vessel temperature decreased to 75°C or lower. The changes in streamlines, speed, and temperature inside the fluid area were accumulated, and the flow of fluid in the gas area was analyzed continuously according to the time dimension.

**FIGURE 2:**
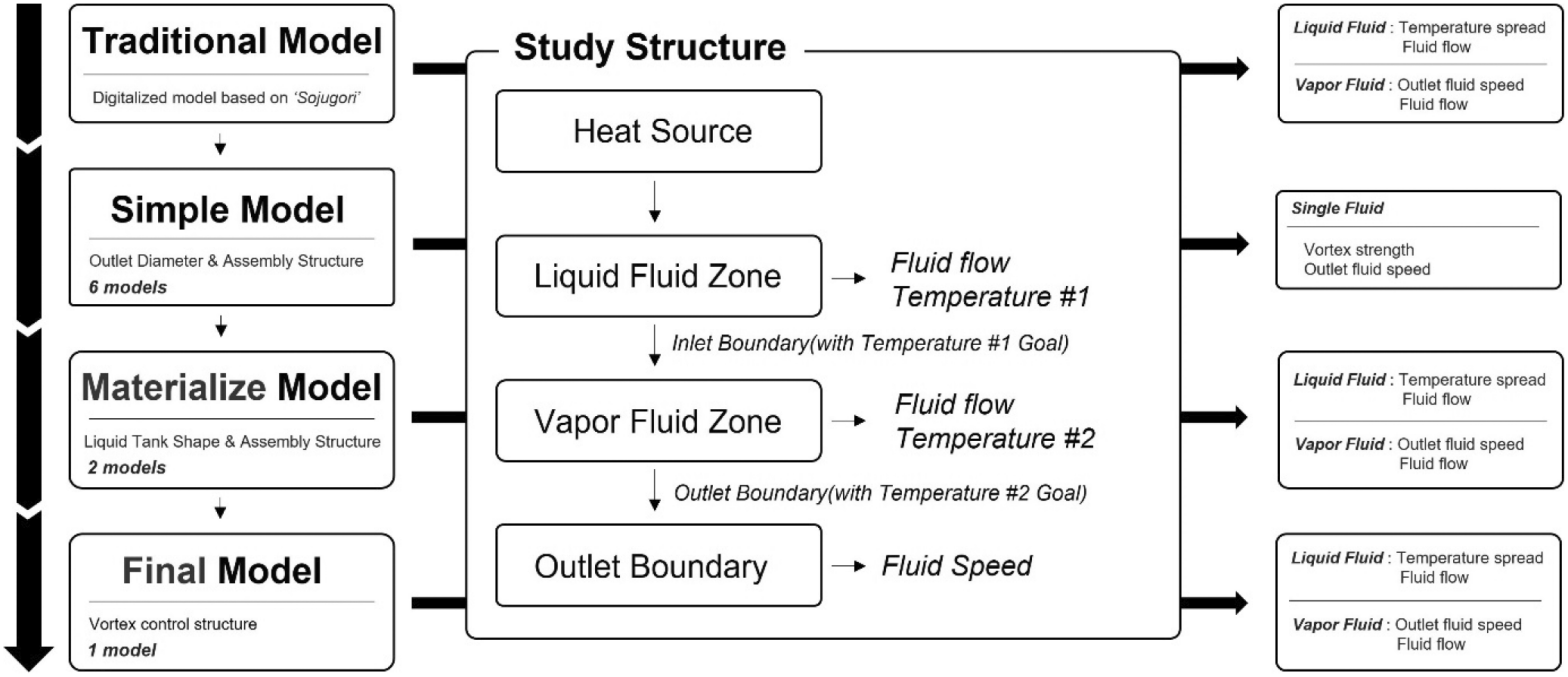
Analysis purpose and flowchart according to the model

In the last step, the residence time, speed, and emission of individual particles were checked by floating objects assumed to be ethanol vapor particles in the fluid flow over time, which was obtained in the second step using the particle study module. The trajectory can be tracked by assuming that ethanol vapor is made up of individual spherical particles of a constant mass and merging the physical particles with the flow analysis result. The analysis condition was set in such a way that particles do not influence the flow, but the flow governs the particle speed and temperature according to density change. The outlet ethanol vapor flows were compared while changing the shape of the distiller using the above analysis method. After the first analysis of the distiller of the traditional shape, six simple models were analyzed to verify the change trend of the internal fluid flow according to the outlet diameter and combination structure. Based on this, two materialized models that can be manufactured with a real earthenware material were designed. The final model was completed by reviewing the problems of fluid flow based on the analysis result of the materialized models and referring to the structural technology for turbulence control. Figure 2 shows the purpose and analysis flowchart of each model. The distillation efficiency of each model was compared by the temperature spread of fermented wash and the outlet ethanol flow speed. In addition, the structure for reflux was designed as a module form and its effects were tracked. Reflux is a phenomenon that a fluid in a distillation process loses energy and recondensed without exiting to the cooler. This is known as a factor that determines the qualities of alcoholic liquors (Claus & Berglund, 2005). It has been reported that the liquor quality can be made soft or heavy by controlling the amount of reflux, and the liquid reinput to the fermented wash also affects the temperature spread (Rodríguez‐Bencomo et al., 2016). Furthermore, in a continuous distiller, the reflux is controlled appropriately using a multilevel mesh network and this process is used for liquor quality control (Matias‐Guiu et al., 2016).

## 3 RESULTS

### 3.1 Analysis result of the Korean traditional distiller *sojutgori*

Many fire points above 100°C were observed in the heating part in the CFD analysis of the digitalized *sojutgori* model. As the flow speed on the inner wall surface decreased sharply, strong vortices were generated at the top and bottom around the center (Figure 1c). Such temperature spread of fermented wash is known to induce the carbonization of organic compounds in the fermented wash and to cause the simultaneous vaporization of water and carbonation component with ethanol (Cha et al., 2020). Furthermore, the vortex that occurs in the movement path of vaporized ethanol interferes with the discharge of ethanol particles and increases the residence time of particles, which acts as an obstacle to the acquisition of homogeneous alcoholic liquors.

### 3.2 Analysis result according to outlet diameter and combination structure

**FIGURE 3:**
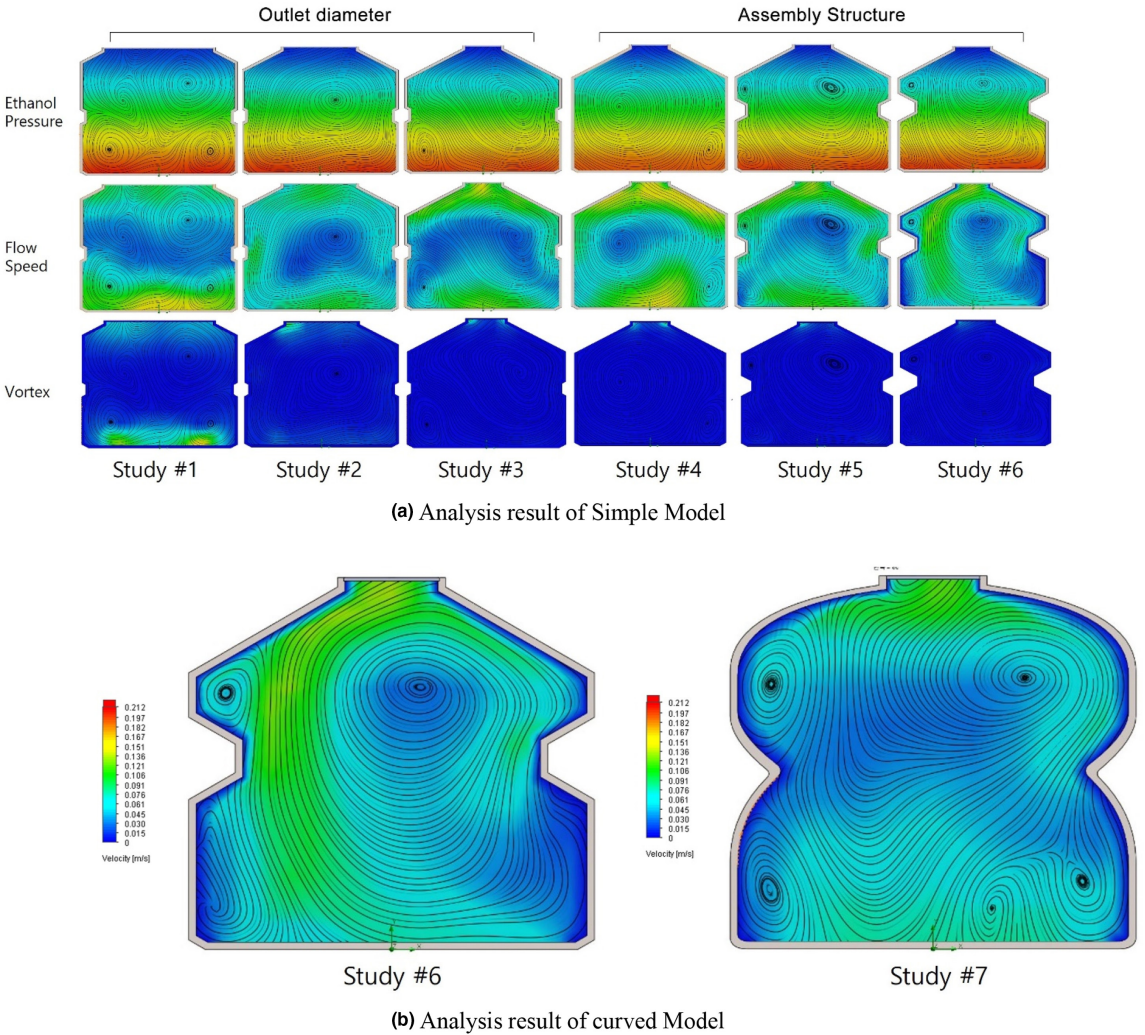
Computational fluid dynamics (CFD) analysis result of the simple and curved model

To infer the meaning of the combination structure that the common shape of *sojutgori* has, simple models with different outlet diameters and combination structures were analyzed. The analysis results are summarized in Figure 3a. A comparison between the analysis results of Study #1 and Study #6 indicates that a simpler container structure strengthens vortex streamlines. Moreover, the smaller the outlet diameter, the faster the flow speed becomes, thus suppressing the formation of vortex. In Study #6, which showed the faster flow speed, the flow speed was faster by up to 74% than that of Study #1.

To track the effect of angled shape of the combination structure on fluid flow, the result of analyzing the model that changed the corners of the container of Study #6, which showed the least vortex occurrences and a fast outlet speed, to curves is shown in Figure 3b. In Study #7, the reduction of flow speed at the inner wall of the container and the frequency of vortex streamline increased.

Particle studies were conducted based on the fluid flow obtained through analysis. In Study #1 with the largest outlet diameter, the average residence time of particles was the shortest. Studies #6 and #7 showed the longest residence time, but the dispersion was the lowest. This is because in the case of Studies #1–5 where a strong vortex occurs, some particles fall in the vortex and cannot escape from it. This dispersion is considered to be a factor that worsens the liquor quality.

### 3.3 Analysis result of materialized models

**FIGURE 4:**
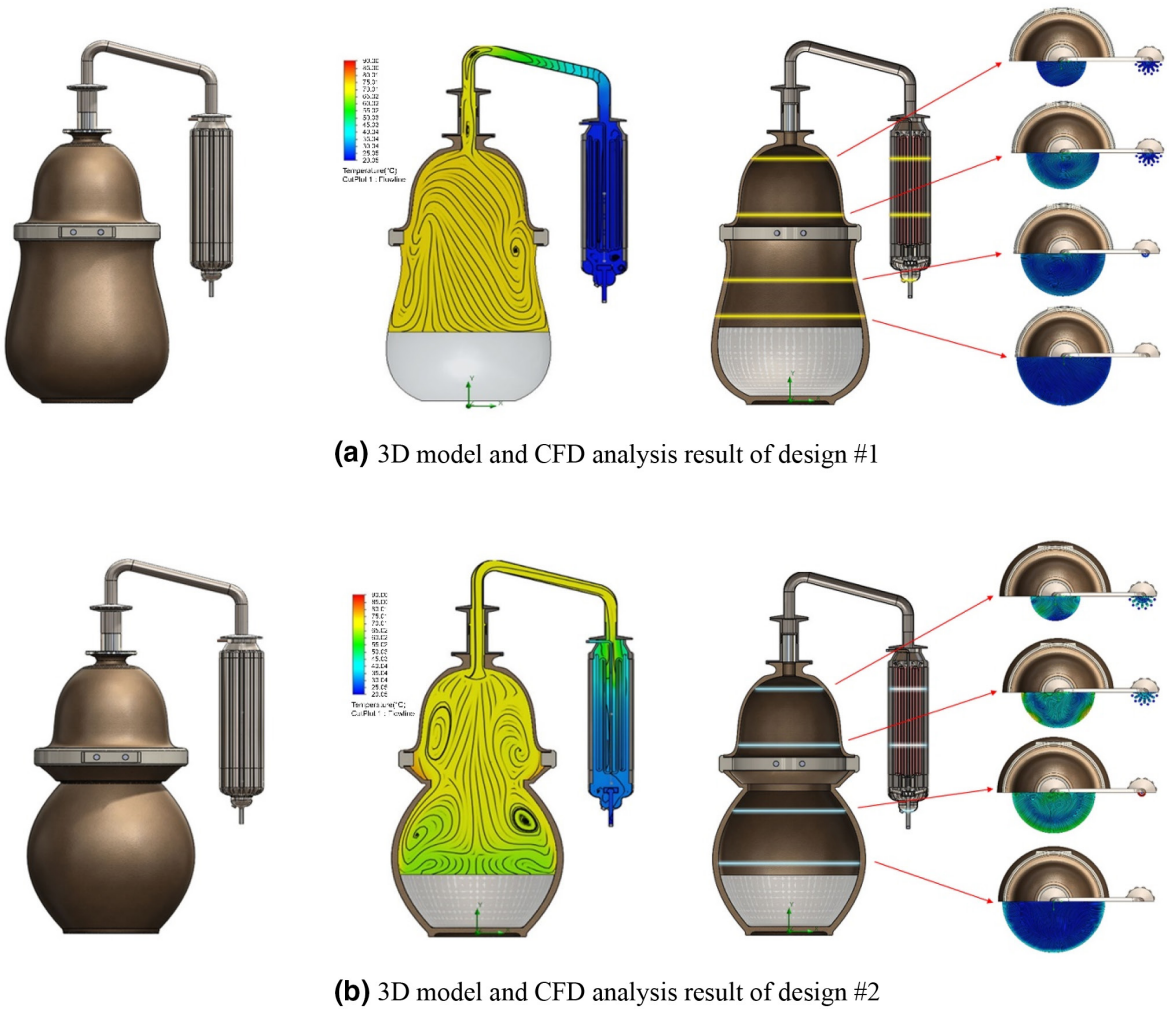
Computational fluid dynamics (CFD) analysis result of the materialized model (Designs #1 and #2)

**FIGURE 5:**
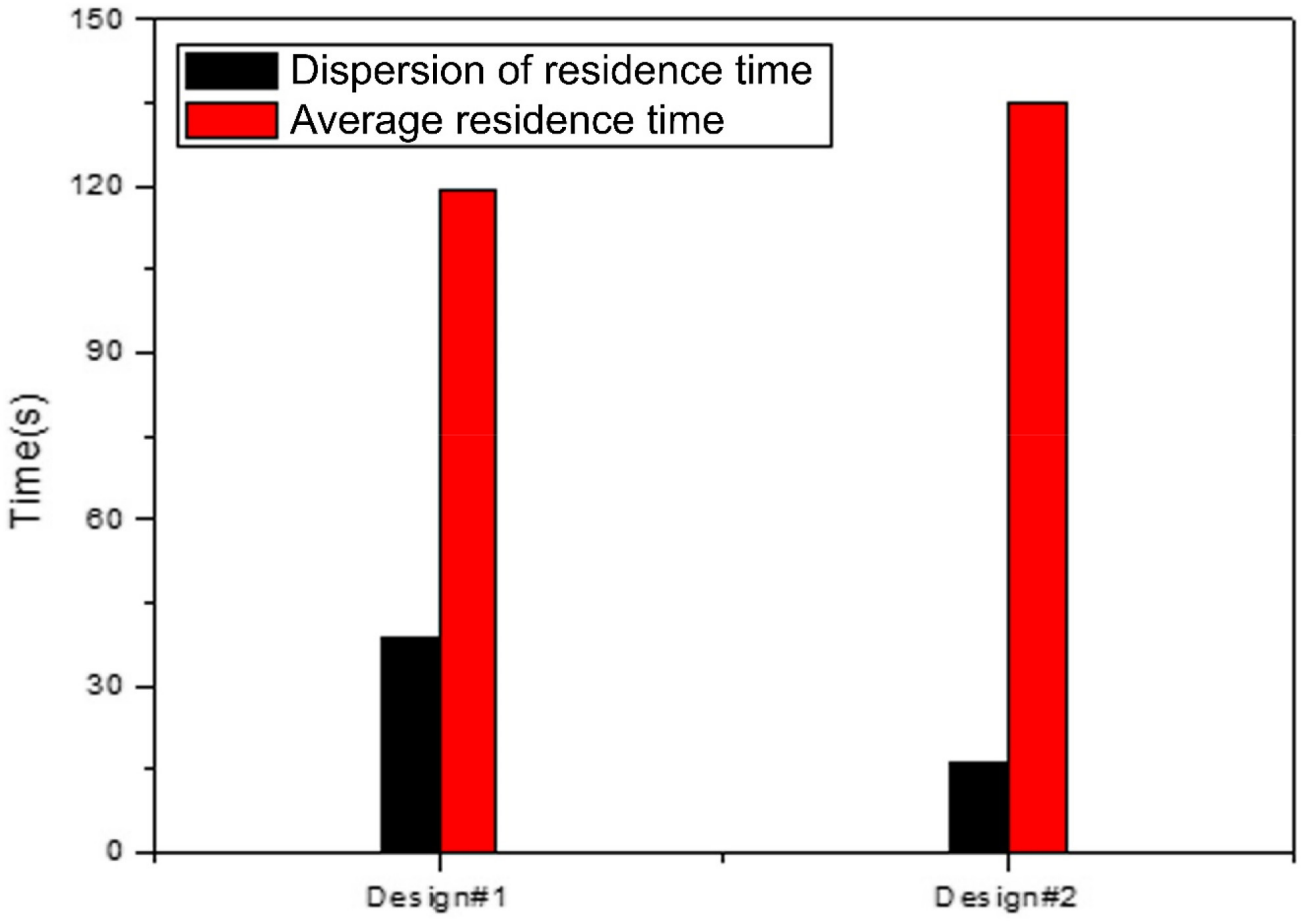
Particle study analysis result of the materialized model

The analysis results of materialized models are shown in Figure 4. Design #1, which has a similar shape as simple model Study #3, is characterized by a wide range of vortexes and a low flow speed. In contrast, Design #2, which has a similar shape as simple model Studies #6 and #7, shows a rapidly increasing flow speed in the center structure where the diameter becomes smaller. Furthermore, stagnation of air flow due to the vortex and a tendency to accumulate thermal energy was observed in the protruding structure. It also shows that some inner gases are circulated for a long time due to the vortex generated symmetrically in the vertical direction. The accelerated flow speed continuously increases to the outlet flow speed, higher by 82% than that of Design #1. The result of particle study is shown in Figure 5. The particle study result of Design #2 is highly similar to the analysis result of *sojutgori*. Similar to the tendency of the simple models, the simpler structure had a shorter average residence time but the residence time dispersion between particles increased.

### 3.4 Effects of blade structure on thermal efficiency in materialized model

Based on Design #2, which showed a lower dispersion of residence time between particles in the materialized model analysis, the final model was analyzed with a blade structure that induces the rotation of the fluid and increases the flow rate to more effectively control the vortex.

**FIGURE 6:**
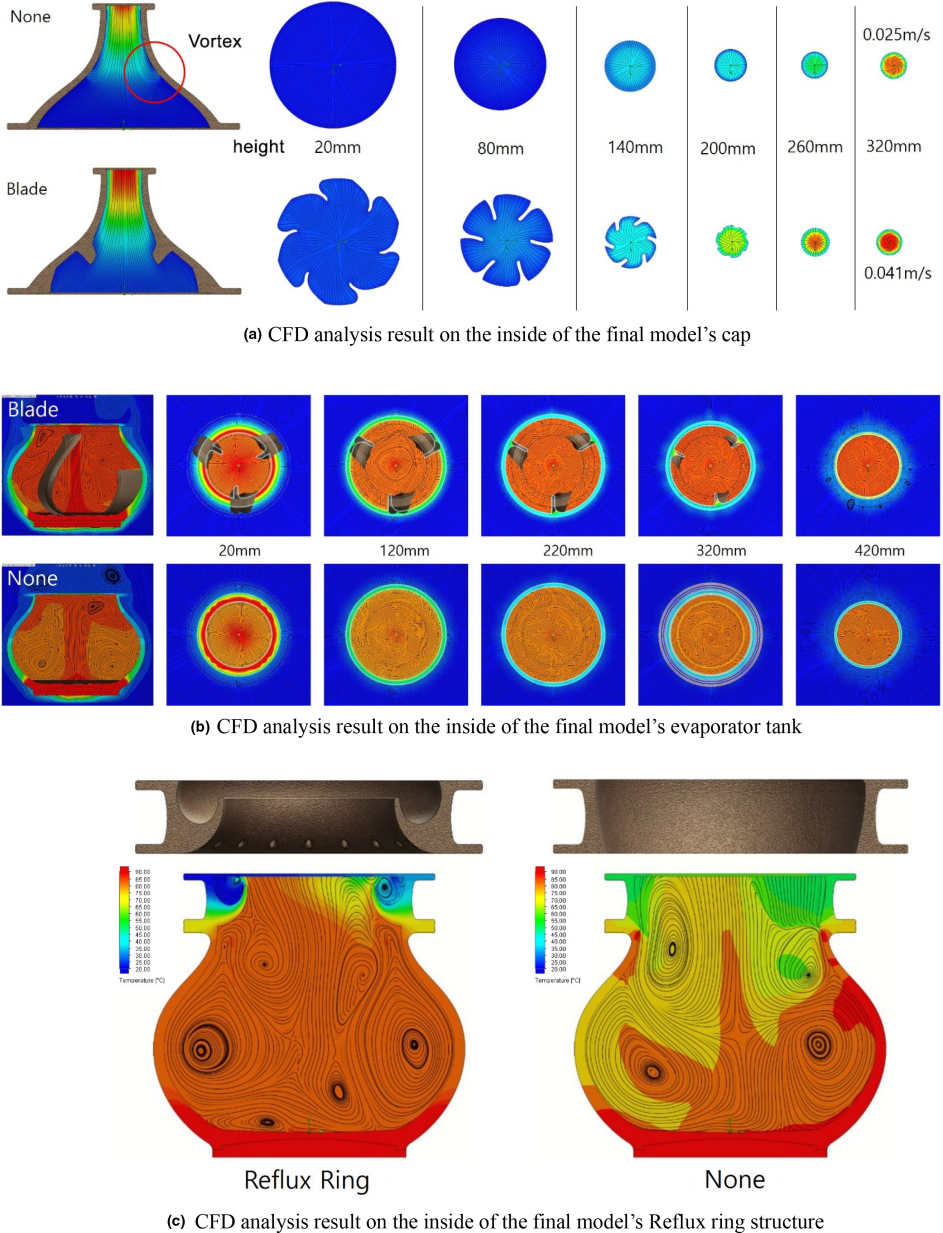
Computational fluid dynamics (CFD) analysis result of the final model

Figure 6b shows the analysis result for the inside of the evaporator tank. To verify the effectiveness of the blade structure, nonblade structures with the same deformity were compared and analyzed. As can be seen in the figure, the fluid has a rotational force due to the blade structure and this promotes a more active convection current. This confirmed that compared to the nonblade type, the vortex frequency decreased and the temperature spread of fermented wash became smaller by 42% on average.

Figure 6a shows the analysis result of the outlet. Regarding the outlet design, to compare the usefulness of blade designed for vortex control, nonblade structures with the same deformity were compared and analyzed. As can be seen from the figure, the blade structures arranged in 60° intervals gave high rotational force to the fluid, and based on this rotational force, vortex on the inner wall was suppressed and a fast discharge speed could be achieved. The final discharge speed was 0.041 m/s in the blade design, approximately 1.8 times faster than 0.025 m/s in the nonblade design.

### 3.5 Analysis result according to heterogeneous material of final model

**FIGURE 7:**
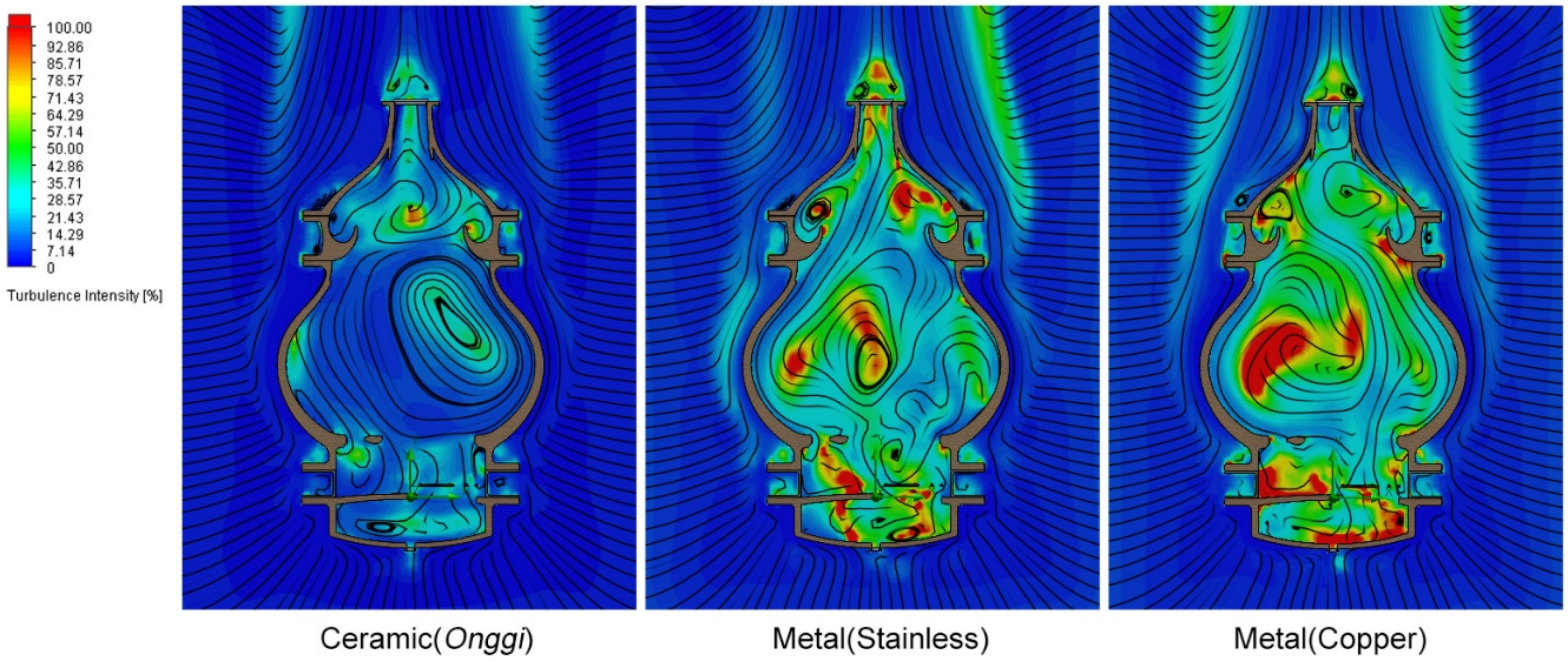
Computational fluid dynamics (CFD) analysis result of internal turbulence intensity according to the material

Significantly different flow properties were observed in the copper and stainless steel materials compared to the earthenware material (Figure 7). This is due to the thermal conductivity of the material. The temperature spread of fermented wash increased and the discharge flow rate decreased due to the rapid heating and accelerated heat loss in the vaporization part. The streamline patterns derived from the same deformation showed a similar tendency, regardless of the change of material. Some fire points at the inner wall exceeded 100°C. It was found that small‐scale vortices increased with the thermal conductivity of the material.

### 3.6 Modern version of modular *sojutgori* with functionality

This study modularized and added a reflux control structure to the final model for which a low temperature spread and a fast discharge speed were predicted through analysis. Through this process, a new structure with a reflux liquid flowing down from the inner side wall and a small amount of fresh fermented wash was designed. The temperature change effect of this structure on fermented wash is shown in Figure 6c. As can be seen in this figure, the liquid that is stagnant in the reflux system heated by the internal temperature is heated again and slowly mixed with fermented wash, thus minimizing the temperature change of fermented wash. The reflux ring structure was found to decrease the average temperature spread by 23%. Furthermore, since this structure is designed as a modular form, it has the functionality that allows stacking multiple modules or changing the diameter of the outlet to enable control of the reflux speed, thus implementing various liquor qualities.

**FIGURE 8:**
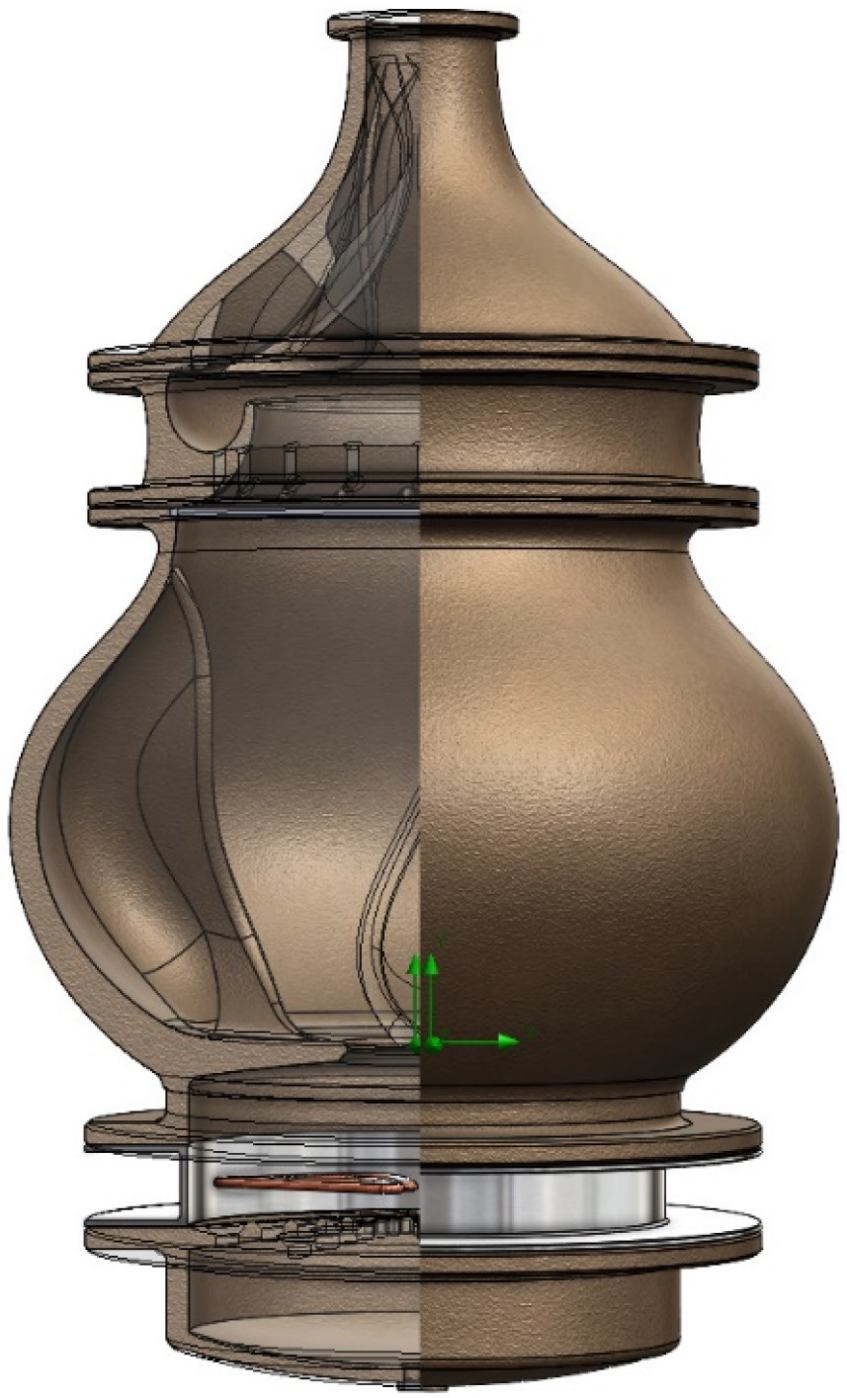
Assembly of the final model

Figure 8 shows the assembly of the final completed design.

## 4 DISCUSSION

### 4.1 Change in temperature spread according to fermented wash container

As a result of estimating various shapes of distillers based on the traditional distiller *sojutgori* and tracking the fluid flow inside them, the fluid flow changed significantly according to the size and frequency of vortex. What is interesting is that more vortices occurred in the design with a large opening and fewer hindrances in the center coupling part, and the shapes became very irregular over time. Consequently, it was observed that the internal fluid flow had an irregular dispersion and the discharge decreased. This appears to be similar vortex control and suppression effects of such structures as winglet, vortex generator, and spoiler, which are artificially installed for vortex control in high‐speed moving objects such as aircrafts and vehicles (Anderson & Gibb, 1998; Jirasek, 2005; Zeng et al., 2010). Such hydrodynamic structures have the same objective as the dimples formed on golf balls. Golf balls with dimples are known to have a 42% higher flying distance than golf balls without them (Li et al., 2015). In other words, through such vortex control effect, a combination structure of the distiller in which the tube diameter is sharply narrowed at the center has a higher flow speed and smaller vortex size, and this induces a direction aligned toward the center. Due to this phenomenon, the dispersion of the residence time of the vaporized ethanol particles is reduced and the total amount of discharge according to time is accelerated. This effect was clearly observed in the two materialized models as well.

### 4.2 Change in flow speed according to outlet shape

The smaller the angle between the outlet's direction vector and the side wall's direction vector, the smaller the frequency of vortices around the outlet was observed. This appears to be because the discharge airflow formed by the pressure change of the fluid with momentum does not match the flow generated by the momentum; as a result, some fluids pass through the outlet and are pushed against and collide with the opposite wall. Considering the intrinsic angular momentum of the streamlines approaching the outlet, the outlet discharge pressure, and the change in tube diameter, the momentum vector should match the outlet vector as much as possible for an efficient outlet structure. This could also be confirmed in the final model designed based on this theory and in the analysis result of materialized model Designs #1 and #2 with an obtuse angle.

### 4.3 Change in distillation efficiency according to distiller material

Stainless steel (15.1 W/m∙k) and copper (401 W/m∙k) materials, which are popular materials for modern distillers, have high thermal conductivity and achieve fast heat exchange with the ambient temperature because of this property. In contrast, the earthenware material with a thermal conductivity below 2.4 W/m∙k showed a high heat shielding rate compared to metals owing to its unique porous structure and low thermal conductivity. Consequently, the earthenware material can control a uniform fermented wash temperature and reduce the recondensation amount due to heat loss. Moreover, it can effectively suppress the change in flow speed around the wall due to temperature loss.

### 4.4 Modern version of modular *sojutgori* with functionality

This study modularized and added a reflux control structure to the final model for which a low temperature spread and a fast discharge speed were predicted through analysis. Through this process, a new structure with a reflux liquid flowing down from the inner side wall and a small amount of fresh fermented wash was designed. The temperature change effect of this structure on fermented wash is shown in Figure 6c. As can be seen in this figure, the liquid that is stagnant in the reflux system heated by the internal temperature is heated again and slowly mixed with fermented wash, thus minimizing the temperature change of fermented wash. The reflux ring structure was found to decrease the average temperature spread by 23%. Furthermore, since this structure is designed as a modular form, it has the functionality that allows stacking multiple modules or changing the diameter of the outlet to enable control of the reflux speed, thus implementing various liquor qualities.

Figure 8 shows the assembly of the final completed design.

## 5 CONCLUSIONS

This study reported the analysis results for a new distiller design that can improve distillation efficiency based on the design of Korean traditional distiller *sojutgori*. Various forms of vortices generated in the distillation process are affected by the temperature change and fluid speed. An appropriate control of vortex decreases the residence time dispersion of ethanol particles and increases the outlet flow speed, thereby discharging effectively vaporized ethanol particles. The optimal design derived based on computer simulation applied the following three key structures that can improve the distillation efficiency based on the analysis result of simple and materialized models.

First, the flow speed of the vaporized fluid was increased by designing a narrowing inlet of the lower tank containing liquid fermented wash, and the convection current speed of the fermented wash with heating was maximized by adding rotational blade structures in 120° intervals to the inner wall. This allowed the continuous mixing of fermented wash and the suppression of temperature spread. The blade structures caused a difference in temperature spread up to 57%.

Second, for the superstructure, the outlet direction vector and the direction vector of the fluid rising through the wall were designed to be less than 18° to control irregular vortices generated around the outlet. In addition, a rotational force was applied to the fluid by adding a streamlined blade structure. The fluid rotating around the outlet effectively reduced the vortex frequency. Moreover, the outlet fluid speed increased 39% on average, and the residence time dispersion of ethanol particles decreased by 78%.

Third, a reflux ring structure module was designed to prevent recondensation due to heat loss at the side wall and the resulting temperature change of fermented wash. As a result, the temperature spread at the top of fermented wash could be effectively suppressed. It was found that the reflux ring structure decreased the temperature spread from the top of fermented wash by 23%.

The best design was achieved to improve the distillation efficiency of each structure. The modular design has the advantages of easy maintenance and expandability to various combinations. It is believed that the data obtained through this study will accelerate the modernization of the Korean traditional distiller *sojutgori*.
